# Disease-Causing Mechanisms and Therapeutic Targets in Infectious Diseases: Implications for Clinical Management and Public Health

**DOI:** 10.3390/biomedicines14030694

**Published:** 2026-03-17

**Authors:** Kristina Sejersen, Susanne Sütterlin, Anders O. Larsson

**Affiliations:** 1 Department of Medical Sciences, Uppsala University, Uppsala University Hospital, 75185 Uppsala, Sweden; anders.larsson@akademiska.se; 2 Unilabs AB, 17154 Stockholm, Sweden; 3 Department of Women’s and Children’s Health, Pediatric Nephrology and Infectious Diseases (PNID) Research Group, Uppsala University, 75185 Uppsala, Sweden; susanne.sutterlin@uu.se; 4 Department of Pediatric Emergency, Uppsala University Children’s Hospital, 75185 Uppsala, Sweden

**Keywords:** infectious diseases, innate immunity, pattern recognition receptors, virulence, antimicrobial resistance, next-generation antimicrobials, host-directed therapy, monoclonal antibodies, chimeric antigen receptor (CAR) immunotherapy, bacteriophages

## Abstract

Infectious diseases remain a major cause of mortality and disability worldwide. This burden is driven, in part, by antimicrobial resistance (AMR) and the re-emergence of epidemic and pandemic threats, underscoring the need for translational research to address knowledge gaps exposed by recent pandemics. Despite significant advances enabled by antibiotics and antivirals, their effectiveness is increasingly constrained by resistance development, limited pathogen spectra, and prolonged development timelines that fail to keep pace with rapidly shifting epidemiology. Diagnostic limitations impede timely pathogen identification and hinder the development of treatment regimens informed by pathogen mechanisms of action. Severe infections frequently involve dysregulated host responses, including hyperinflammation, inflammasome activation, and endothelial or immunothrombotic injury, which may progress to sepsis, immunoparalysis, or chronic sequelae, highlighting the limitations of pathogen-centered paradigms. Conventional biomarkers and culture-based microbiology are often slow or nonspecific, while molecular assays may not reliably distinguish colonization from active infection or capture host-response heterogeneity shaped by age, immune competence, and disease stage. This review synthesizes mechanistic and translational insights across three interrelated axes: (i) host–pathogen interactions, with a focus on innate immune sensing networks (e.g., Toll-like receptors, inflammasomes, RIG-I-like receptors, and cGAS-STING) and microbial replication and immune evasion strategies; (ii) clinical and public health implications, spanning acute organ dysfunction syndromes, post-acute infection syndromes, and AMR-driven health system strain; and (iii) emerging therapeutics along a continuum of pathogen-, virulence-, host-, and immune-directed approaches. Emphasis is placed on anti-virulence therapeutics, bacteriophage therapy, monoclonal antibodies, and engineered immune modalities within frameworks of quantitative translational pharmacology and implementation science. Finally, an integrative conceptual framework encompassing mechanistic phenotypes, host-response diagnostics, and stage-adapted therapeutic combinations is proposed to guide rational intervention across endemic infections and future pandemic preparedness.

## 1. Introduction

### 1.1. Global Burden and Unmet Needs

Infectious diseases continue to be among the foremost causes of mortality and overall disease burden worldwide, despite substantial progress in the domains of prevention, diagnostics, and therapeutics [1,2,3]. Bacterial infections account for a substantial proportion of this mortality and are linked to both common and severe clinical syndromes, including pneumonia, meningitis, tuberculosis, and a wide range of soft-tissue and gastrointestinal infections. These infections may lead to sepsis and other forms of severe infection-associated organ dysfunction [1,2,3].

Over the past two decades, recurrent epidemics and pandemics—culminating in the emergence of the coronavirus disease 2019 (COVID-19) pandemic—have exposed critical vulnerabilities in global preparedness, healthcare infrastructure, diagnostic capacity, and therapeutic development pipelines [4,5,6]. These events have demonstrated the rapid emergence and re-emergence of pathogens capable of overwhelming healthcare systems, causing profound clinical, societal, and economic disruption, and exacerbating existing challenges in infectious disease management [4,5,6,7].

Antibiotics and antivirals have been instrumental in improving clinical outcomes. However, antimicrobial resistance (AMR), a limited spectrum of pathogens and slow development cycles are posing growing challenges. Nevertheless, the outcome of several severe infections is not primarily determined by antimicrobial failure, but by pathogen virulence, the host’s response and timely supportive interventions. This has led to a significant unmet need for new therapeutic strategies in the treatment of infectious diseases [8,9,10]. The extensive empirical utilization of antimicrobials during epidemics and pandemics has further augmented selective pressure, expediting the emergence and global propagation of resistance [9,10]. Beyond the individual patient outcomes, severe infections and AMR exert substantial public health and societal impacts, including straining healthcare systems, economic loss, and widening global health inequities [1,2,3,10].

### 1.2. Mechanistic Foundations of Host–Pathogen Interactions

The defense of the host against infection is mediated by a highly coordinated network of cellular and molecular immune pathways. These pathways detect invading pathogens while maintaining tissue homeostasis [11,12]. The innate immune system serves as the primary line of defense, relying on pattern-recognition receptors (PRRs) to detect pathogen-associated and damage-associated molecular patterns (PAMPs and DAMPs), thus prompting the swift activation of antimicrobial and inflammatory programs [11,12].

Cytosolic DNA sensing pathways, most notably the cyclic GMP-AMP synthase (cGAS)–stimulator of interferon genes (STING) axis, detect microbial or aberrantly localized self-DNA and trigger type I interferon production and inflammatory signaling [13,14]. In concert with Toll-like receptors (TLRs), NOD-like receptors (NLRs), inflammasomes, and RIG-I-like receptors (RLRs), these pathways integrate microbial sensing with nuclear factor κB (NF-κB) and interferon regulatory factor activation, thereby shaping early innate responses and downstream adaptive immunity [11,12,15]. These sensing and effector pathways operate within a host context that is shaped by immune regulation, tissue integrity, and the balance between antimicrobial defense and immunopathology. This contributes to substantial variability among individuals in the control of early pathogens and the subsequent development of disease [11,12,15].

It is imperative to acknowledge the significance of immune mechanisms in the regulation of pathogens. Dysregulation of these mechanisms, however, can precipitate excessive systemic inflammation, immunopathology, tissue injury, and organ dysfunction. These phenomena are pivotal in the pathogenesis of severe infections and pandemic-associated critical illness [16,17,18]. Sepsis, defined by the Sepsis-3 consensus as life-threatening organ dysfunction caused by a dysregulated host response to infection, represents a unifying clinical syndrome linking bacterial infections, viral pandemics, and immune-mediated tissue damage [18]. According to global estimates, sepsis is associated with millions of deaths each year. This has led the World Health Organization (WHO) to recognize sepsis as a global health priority, underscoring the urgent need for improved prevention, early diagnosis, and personalized therapeutic strategies [19,20].

### 1.3. Diagnostic Limitations and Etiological Heterogeneity

Timely and accurate diagnosis is critical to reducing delays between symptom onset and appropriate management. This is important because it can lower the risk of progression to severe disease, sepsis, and death. It can also minimize unnecessary antimicrobial exposure [21,22,23]. In routine clinical practice, the initial distinction between bacterial and non-bacterial infections relies heavily on clinical assessment supported by non-specific biomarkers, such as leukocyte count and C-reactive protein. However, these biomarkers lack sensitivity and specificity, and fail to identify a substantial proportion of bacterial infections [21,23].

Microbiological confirmation has traditionally relied on blood culture-based methods, which require 24–72 h for results. A substantial proportion of sepsis cases yield negative blood cultures, a finding not only attributable to prior antimicrobial exposure, sampling limitations, or fastidious and slow-growing pathogens, but also to biological factors such as rapid intravascular clearance, low-level or transient bacteremia, and focal infections without continuous bloodstream involvement [22]. Advancements in molecular diagnostics, encompassing pathogen-specific and syndromic polymerase chain reaction (PCR)-based assays, have led to significant enhancements in the detection of viral and bacterial pathogens. These advancements have concomitantly reduced the time to diagnosis, a benefit that has been most evident in the management of respiratory and bloodstream infections [24,25,26]. However, these approaches are constrained by several factors. First, the target panels are limited in scope. Second, there is an inability to reliably distinguish colonization from active infection. Third, there is incomplete correlation with disease severity or host inflammatory responses [25,26,27].

The aforementioned diagnostic challenges are further compounded by the heterogeneity of infectious etiologies, which may present with overlapping clinical phenotypes but engage distinct host immune pathways. Such etiologies may include bacterial and viral infections [15,28]. The collective presence of these limitations has prompted the development of adjunct biomarkers, such as procalcitonin and host-response signatures, with the objective of enhancing early etiological differentiation and facilitating more precise therapeutic decision-making, particularly during pandemics and periods of healthcare system strain [21,28].

### 1.4. Mechanistic Insights into Host—Pathogen Interactions, and Its Clinical and Public Health Implications

The limitations of pathogen-centered diagnostics and classical antimicrobials have led to increased interest in therapeutic strategies that extend beyond direct pathogen killing. A range of approaches is currently being explored in the fight against pathogens. These include virulence-directed approaches, host-directed therapies aimed at modulating immune and metabolic pathways, bacteriophage-based interventions, and antibody-mediated strategies designed to neutralize pathogens or toxins without exacerbating selective pressure for resistance [28].

**Figure 1:**
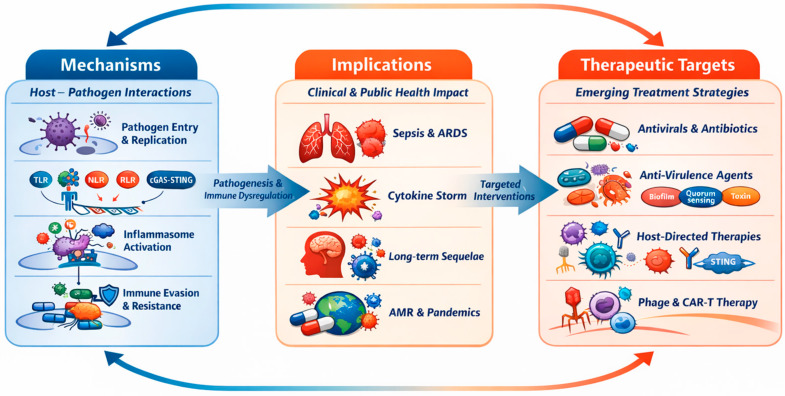
Conceptual framework linking mechanisms, clinical implications, and therapeutic targets in infectious diseases. Schematic overview illustrating how host–pathogen interactions and innate immune sensing mechanisms drive clinical outcomes such as sepsis, ARDS, and long-term sequelae, and how these processes inform pathogen-, virulence-, and host-directed therapeutic strategies. Arrows indicate the dynamic and bidirectional relationships among mechanisms, disease manifestations, and intervention points across acute infection, critical illness, and post-acute phases.

In light of this, the present review synthesizes mechanistic insights into host–pathogen interactions, paying particular attention to clinical and public health implications. It methodically examines emerging therapeutic targets and strategies for infectious diseases in the era of antimicrobial resistance and pandemics. The review is organized around three interconnected axes: mechanisms, encompassing molecular and cellular processes governing infection; implications, addressing clinical outcomes and broader public health consequences; and therapeutic targets, spanning pathogen-, virulence-, host-, and immune-based intervention strategies. The primary objective of this review is to synthesize mechanistic and translational insights into host–pathogen interactions, innate immune sensing, and virulence- and host-directed therapies, and to integrate these insights into a conceptual framework for stage-adapted diagnostics and treatment strategies applicable to both endemic infections and future pandemics (Figure 1).

## 2. Mechanisms in Infectious Diseases

### 2.1. Innate Immune Sensing and Dysregulation

Innate immunity constitutes the primary line of defense against invading pathogens. It relies on germline-encoded PRRs to detect conserved PAMPs and DAMPs, thereby rapidly initiating host defense mechanisms [11,12]. Upon sensing microbial products or perturbed self-structures, membrane-bound and endosomal TLRs, C-type lectin receptors (CLRs), and soluble PRRs act in concert with cytosolic sensors such as NLRs and inflammasomes, RLRs, and the cGAS-STING pathway. As demonstrated in the relevant literature, these sensing pathways converge on NF-κB, mitogen-activated protein kinase (MAPK), and interferon regulatory factor (IRF) signaling. This convergence results in the induction of pro-inflammatory cytokines, type I and type III interferons (IFNs), chemokines, and inflammatory forms of cell death [11,12,15,29].

It has been established that TLRs, CLRs, and soluble PRRs are of particular importance for the early control of extracellular bacteria, fungi, and a multitude of viruses. Conversely, inflammasome-forming NLRs such as NLRP3, NLRC4 and NLRP1, the DNA sensor AIM2, RLRs, and the cGAS-STING pathway primarily detect intracellular pathogens or misplaced self-nucleic acids. It has been demonstrated that innate immune sensors contribute to the elimination of pathogens and the shaping of adaptive immunity. The mechanisms by which these sensors influence adaptive immunity include Th1 and Th17 polarization, germinal center formation, and the development of durable antibody responses [15,30].

While the precise regulation of PRR activation is imperative for optimal host defense, the loss of temporal or spatial control over these pathways profoundly alters their biological outcome. Excessive or chronic activation of innate immune sensors has been demonstrated to drive systemic hyperinflammation and cytokine storm [16,31], leading to acute respiratory distress syndrome (ARDS) and sepsis during severe infections, including but not limited to cases of severe acute respiratory syndrome (SARS) [16,17,18,31,32,33] and influenza [32,33]. Clinical and immunological studies in patients with severe cases of SARS-CoV-2 infection have demonstrated a dysregulation of innate immune activation, characterized by excessive cytokine production, blunted or delayed early type I interferon responses, and sustained inflammasome activity. These factors contribute to tissue damage and poor clinical outcomes [31,32,33]. In addition to acute infection, sustained inflammasome activation, dysregulated interferon production, and amplified sensing of DAMPs released during tissue injury promote the transition from acute to chronic inflammation. This, in turn, contributes to immune-mediated inflammatory diseases [14,15,34,35,36,37].

This dual role of innate immune sensing has fueled major efforts to therapeutically target PRR pathways. Depending on the clinical context, the development of agonists is underway for the purpose of enhancing antimicrobial defense. These agonists may be utilized as vaccine adjuvants or antiviral immunotherapies. In contrast, antagonists and pathway inhibitors are being investigated as potential therapeutic agents to mitigate hyperinflammation and immunopathology in sepsis, severe viral pneumonia, and chronic inflammatory disorders [36,38,39]. An overview of canonical innate immune sensors, their roles in infectious diseases, and current therapeutic strategies is provided in Table 1.

Building on these innate sensing pathways, we next consider how pathogens—illustrated here with a viral exemplar—enter host cells, replicate, and evade early immune control.

### 2.2. Pathogen Entry and Replication (Viral Focus)

This section uses SARS-CoV-2 as an example to illustrate the general principles of viral entry, replication, immune evasion, and therapeutic targeting applicable to other emerging and pandemic-prone viruses.

#### 2.2.1. Viral Entry and Uncoating

SARS-CoV-2 is an enveloped, positive-sense RNA virus whose trimeric spike (S) glycoprotein is responsible for binding to the host cell receptor angiotensin-converting enzyme 2 (ACE2) and initiating entry via plasma membrane fusion or endocytosis [54,55,56]. Structural studies have demonstrated that the receptor-binding domain (RBD) within the S1 subunit engages ACE2 with high affinity. In addition, the prefusion conformation of the spike underpins neutralizing antibody recognition and vaccine design [54,55].

The proteolytic activation of the S at the S1/S2 and S2′cleavage sites by host proteases—most notably TMPRSS2 at the cell surface and, alternatively, endosomal cathepsins—exposes the S2 fusion machinery and enables the merger of the viral and host membranes [56,57]. The presence of a multibasic, furin-cleavable S1/S2 site has been demonstrated to further enhance entry efficiency and infection of human lung cells [57]. Subsequent to fusion, the nucleocapsid is released into the cytoplasm, and the viral RNA genome is uncoated. This genome then functions as mRNA for the translation of the replicase polyproteins pp1a and pp1ab [6].

#### 2.2.2. Polyprotein Processing and Replication–Transcription Complex Formation

The pp1a and pp1ab polyproteins are subject to proteolytic processing by two virally encoded cysteine proteases: the main protease (Mpro/3CLpro) and the papain-like protease (PLpro). This process yields non-structural proteins (nsps) that subsequently assemble into the replication–transcription complex (RTC) [6,58,59,60]. Mpro has been shown to cleave polyproteins at multiple conserved sites, thereby liberating NSPs that are essential for RNA synthesis and RTC architecture [59]. Concurrently, PLpro processes the N-terminal region of the polyprotein and antagonizes host antiviral signaling through deubiquitination and deISGylation of key host factors [58,60].

Research has demonstrated that RTC activity occurs on virus-induced double-membrane vesicles derived from intracellular membranes. These membranes spatially organize viral RNA synthesis and partially shield replication intermediates from innate immune sensing [6]. The core RNA synthesis machinery comprises the RNA-dependent RNA polymerase (RdRp; nsp12) in complex with cofactors nsp7 and nsp8 [61,62]. Cryo-electron microscopy structures have resolved both the polymerase core and an actively replicating complex, providing a mechanistic basis for inhibition by nucleotide analogues [61,62]. It has been established that additional non-structural proteins (NSPs) function in a coordinated manner to regulate critical aspects of the viral lifecycle. This includes the nsp13 helicase, the nsp14 3′–5′ exonuclease (ExoN), which plays a crucial role in proofreading, and capping enzymes such as nsp14 and nsp16. These NSPs collaborate to orchestrate processes such as RNA synthesis, replication fidelity, and the process of mRNA-like capping of viral transcripts [6].

The structural proteins spike (S), envelope (E), and membrane (M) are synthesized and inserted into the endoplasmic reticulum. Thereafter, they are trafficked to the ER–Golgi intermediate compartment, where they undergo assembly with nucleocapsid (N)-encapsidated genomic RNA. Subsequent to this, progeny virions are released via the secretory pathway [6,63].

#### 2.2.3. Innate Immune Sensing, Antagonism and Evasion

Viral RNA species and replication intermediates generated during SARS-CoV-2 infection can be detected by host pattern-recognition receptors, including RLRs and endosomal TLRs. These receptors activate signaling cascades that induce type I and III interferon responses and downstream interferon-stimulated genes (ISGs) [6,31,45]. SARS-CoV-2 encodes a multitude of proteins that potently antagonize these innate immune pathways.

Nsp1 functions as a host shutoff factor by obstructing the mRNA entry channel of the 40S ribosomal subunit, resulting in global suppression of host translation and antiviral gene expression [64]. PLpro has been shown to have a further inhibitory effect on innate immune signaling by removing ubiquitin and ISG15 from key adaptor proteins. This results in a blunting of interferon induction and amplification [58,60]. The interplay among efficient receptor engagement and entry, rapid RTC formation, and multifaceted antagonism of innate immune defenses facilitates high-level viral replication. In susceptible individuals, delayed or dysregulated immune activation contributes to hyperinflammation, ARDS, multi-organ involvement, and severe clinical disease [31,33,52].

In consideration of the aforementioned elements, it is evident that the SARS-CoV-2 virus employs a multifaceted approach to its replication and transcription processes. This approach involves the coordinated exploitation of various mechanisms, including the entry of the virus into the host cell, the subsequent processing of the viral polyprotein by the host’s protease, and the assembly of the replication–transcription complex. In addition to these mechanisms, the virus deploys a multitude of antagonists of innate antiviral signaling, thereby evading the immune response of the host. These mechanistic insights have directly informed antiviral and host-directed therapeutic strategies. Table 2 is a compendium of viral and host targets, their mechanistic roles, representative therapeutic agents, and key clinical trial outcomes together with the predominant level of supporting evidence.

### 2.3. Bacterial Virulence and Antimicrobial Resistance

The development of bacterial pathogenicity is facilitated by the coordination of virulence programs, which enable the following: the colonization of hosts, the evasion of immune systems, and the subsequent damage of tissues. It has been established that the core virulence mechanisms of bacteria include the formation of biofilms, the regulation of gene expression by quorum sensing (QS), the production and secretion of toxins, surface adhesins, two-component regulatory systems (TCSs), and cyclic di-GMP signaling pathways [77,78,79,80,81]. These determinants are embedded in global regulatory networks that integrate environmental cues and population density, allowing pathogens to dynamically adapt their behavior during infection.

Biofilms represent a central virulence strategy in many clinically relevant bacteria. Biofilm-embedded cells are encased in an extracellular matrix composed primarily of extracellular DNA (eDNA), proteins, and polysaccharides. This matrix confers pronounced tolerance to host immune defenses and antimicrobial agents compared with planktonic cells [77,80]. The initial attachment of bacteria to surfaces is facilitated by surface adhesins, including pili, fimbriae, and sortase-anchored proteins. These adhesins promote stable interactions with host tissues and abiotic surfaces, thereby facilitating the initiation of biofilms [77,79]. As biofilms mature, matrix components impede antimicrobial penetration and generate physicochemical gradients that further reduce antibiotic efficacy, contributing to chronic, relapsing, and device-associated infections.

Concurrently, numerous pathogens secrete a wide array of toxins, encompassing classical A/B exotoxins and effector proteins that are conveyed via specialized secretion systems. These virulence factors directly damage host tissues, modulate innate immune responses, and contribute to systemic disease manifestations [78,79,81]. Virulence factor production is known to be energetically costly and can expose bacteria to immune recognition. For this reason, its expression is typically tightly regulated.

The global control of virulence is mediated by interconnected regulatory systems, most notably QS, TCSs, and cyclic di-GMP signaling [77,79,81,82]. QS has been demonstrated to facilitate bacterial perception of population density through the action of autoinducer molecules, thereby orchestrating a coordinated expression of virulence genes across the community, the maturation of biofilms, and the production of toxins [77,79,81]. TCSs have been shown to link specific environmental or host-derived cues to transcriptional programs controlling adhesins, toxins, and secretion systems [77,79,81]. Cyclic diguanylate monophosphate (c-di-GMP) serves as a second messenger, orchestrating the transition between motile and sessile lifestyles. Elevated intracellular c-di-GMP levels generally promote biofilm formation and reduced motility, whereas lower levels favor dispersal and planktonic growth [77,79,81]. Collectively, these regulatory networks ensure the appropriate spatial and temporal expression of virulence programs during the course of an infection.

AMR emerges through a variety of molecular mechanisms that often coexist within the same bacterial strain [83,84]. A number of mechanisms have been identified as playing a role in this phenomenon. These include active efflux pumps, which have been shown to reduce intracellular antibiotic concentrations. Enzymatic drug inactivation, such as that caused by β-lactamases and aminoglycoside-modifying enzymes, is another such mechanism. Target modification, for example through altered penicillin-binding proteins, mutations in DNA gyrase/topoisomerase, or RNA polymerase, is also of relevance. Finally, reduced permeability due to porin loss or outer membrane remodeling, particularly in Gram-negative pathogens, has been demonstrated as well [83,84]. The global emergence of multidrug-resistant and extensively drug-resistant bacteria has been driven by the accumulation and horizontal dissemination of resistance determinants, resulting in increased morbidity, mortality, and healthcare costs [84].

Conventional antibiotics primarily target essential cellular processes, including cell wall synthesis, protein synthesis, DNA replication, and folate metabolism. While this method has proven to be highly effective, it has also exerted significant selective pressure based on survival, thereby facilitating the rapid evolution and dissemination of resistance determinants [8,84]. These evolutionary dynamics have motivated a conceptual shift toward therapeutic strategies that attenuate virulence rather than directly inhibiting bacterial growth.

Next-generation antimicrobials (NGAs) are therefore commonly defined as anti-infective agents that selectively interfere with bacterial virulence mechanisms. These mechanisms include biofilm integrity, adhesin function, toxin activity, and global regulatory pathways. NGAs are administered at concentrations that do not inhibit viability [77,81]. The objective of NGAs is to disarm pathogens rather than to kill them. This approach is intended to reduce their pathogenic potential, enhance the immune clearance mechanisms of the host, and potentiate the activity of conventional antibiotics. Theoretically, this method exerts less survival-based selective pressure and thereby slows resistance evolution [77,81]. As outlined in Table 3, a comprehensive overview of pivotal classes of NGAs targeting bacterial virulence is provided, accompanied by a de-tailed exposition of their mechanisms of action, the clinical contexts in which they are applicable, and the predominant levels of supporting evidence available [77,81].

## 3. Implications of Infectious Diseases

### 3.1. Clinical Implications

Infectious diseases manifest in a wide range of clinical presentations, ranging from mild, self-limited illnesses to severe pneumonia, ARDS, multiorgan failure, and death [7].

In the initial phase of an infection, tissue injury is caused by both direct pathogenic effects on cells and an excessive and dysregulated immune response in the host [85].

In severe viral infections such as SARS or severe COVID-19 caused by SARS-CoV-2, this hyperinflammatory state may culminate in a hyperinflammatory spectrum often described as a cytokine storm-like syndrome. A cytokine storm is characterized by markedly elevated levels of pro-inflammatory mediators, including interleukins (IL-2, IL-6, IL-7), granulocyte colony-stimulating factor, interferon-γ–inducible protein 10, and tumor necrosis factor-α [16].

Cytokine-driven hyperinflammation is a central contributor to organ dysfunction in severe infection and sepsis [86].

This systemic inflammatory response has been demonstrated to contribute directly to pulmonary injury, vascular leakage, circulatory collapse, and multiorgan dysfunction [87].

A fundamental aspect of infection-driven immunopathology is the activation of inflammasomes [15].

Inflammasomes are cytosolic multiprotein complexes that are assembled by pattern-recognition receptors upon detection of pathogen-associated molecular patterns or endogenous danger signals [15].

The activation of these receptors leads to the initiation of inflammatory caspase signaling, which in turn results in the maturation and secretion of IL-1β and IL-18. Additionally, this process induces pyroptosis, a form of programmed cell death characterized by significant inflammation [88].

Although inflammasome activation is critical for effective host defense, excessive or sustained signaling can lead to tissue injury and has been associated with the pathogenesis of both infectious and sterile inflammatory diseases [89].

At the severe end of the clinical spectrum, dysregulated host responses converge into sepsis, a unifying clinical syndrome defined by the Sepsis-3 consensus as life-threatening organ dysfunction caused by infection [18].

Clinically, sepsis is identified by an acute increase in the Sequential Organ Failure Assessment (SOFA) score of at least two points, which is associated with an in-hospital mortality exceeding 10% [18].

Septic shock is the most severe condition on the clinical spectrum of sepsis. It is characterized by severe circulatory and metabolic abnormalities, including hypotension and elevated serum lactate. It is associated with a very high mortality rate across all age groups, often exceeding 30–40% in many cohorts [18]. These observations emphasize the important role of host-mediated injury in determining clinical outcomes, rather than pathogen burden alone [90]. It is notable that the host response to severe infection and sepsis is often biphasic, featuring an initial hyperinflammatory phase followed by prolonged immunosuppression [91]. This state, known as sepsis-induced immunoparalysis, is characterized by lymphocyte apoptosis, impaired antigen presentation, T-cell exhaustion, and an increased susceptibility to secondary infections and viral reactivation [91].

As indicated by the existing literature, persistent immune dysfunction has been associated with adverse long-term outcomes and increased late mortality among sepsis survivors [90].

Importantly, these trajectories are modified by baseline host factors, such as age and immune competence. Children may exhibit different immune set points and clinical phenotypes. On the other hand, older adults and immunosuppressed patients are predisposed to atypical presentations, impaired pathogen clearance, an increased risk of secondary infections or viral reactivation, and prolonged immune dysfunction [17,90,91].

The recognition of this dynamic immune trajectory is of significant clinical importance, as indiscriminate immunosuppression may prove deleterious in later stages of the disease. This underscores the necessity of immune monitoring and stage-adapted therapeutic strategies [91].

A significant number of survivors of severe infectious diseases experience long-term sequelae and post-infectious syndromes in the aftermath of the acute phase [92]. Although post-COVID-19 conditions have drawn renewed attention to this issue, these manifestations form part of a wider group of post-infectious conditions collectively known as post-acute infection syndromes (PAIS). These syndromes are characterized by non-specific yet debilitating symptoms such as fatigue, neurocognitive impairment and muscle weakness following various infections [92]. These syndromes have been observed to persist for extended periods, ranging from months to years, and have been shown to result in a significant decline in quality of life. This decline is indicative of intricate host–pathogen and immune regulatory interactions [92].

Critically ill patients are also susceptible to post-intensive care syndrome (PICS), a condition characterized by the emergence or exacerbation of impairments in physical, cognitive, and mental health following an intensive care unit (ICU) stay [93].

PICS has been demonstrated to impact a substantial proportion of ICU survivors, exerting a considerable effect on long-term functional status and quality of life [94].

The impact of PICS extends to family members and caregivers, underscoring the necessity for structured follow-up and multidisciplinary rehabilitation programs in the post-ICU period [93].

In patients with severe SARS-CoV-2 infection (COVID-19), in many ICU and hospitalized cohorts, more than half develop at least one post-acute complication, with myopathy, dysphagia, and pressure injuries among the most frequently reported [95].

The mounting recognition of post-acute and chronic manifestations, including post-acute sequelae of SARS-CoV-2 infection (PASC), underscores the necessity for structured long-term follow-up, multidisciplinary rehabilitation, and the integration of survivorship care into infectious disease management [95].

Collectively, these observations underscore the notion that clinical outcomes in infectious diseases are shaped not only by the characteristics of the pathogen but also by the magnitude, duration, and regulation of the host immune response [85].

An enhanced comprehension of immunopathological mechanisms, including but not limited to cytokine storm, inflammasome activation, and immune exhaustion, furnishes a framework for the development of targeted immunomodulatory interventions and risk stratification based on biomarkers [17].

### 3.2. Public Health and Societal Implications

Pandemics and AMR represent converging global health threats that substantially contribute to morbidity, mortality, and sustained strain on healthcare systems worldwide [1].

Together, they threaten past gains in infectious disease control and undermine the resilience, equity, and sustainability of health systems, with disproportionate impacts in low- and middle-income countries (LMICs) [96].

#### 3.2.1. Global Morbidity and Mortality

According to Global Burden of Disease analyses, AMR was directly responsible for approximately 1.27 million deaths and was associated with nearly 4.95 million deaths globally in 2019. This places AMR among the leading infectious causes of death worldwide [1].

Complementary analyses of pathogen-specific mortality further underscore the scale of infection-related mortality and its interaction with resistance patterns [2].

Updated modeling studies indicate that, in the absence of intensified and coordinated control measures, there is a high probability of a substantial increase in AMR-attributable mortality over the coming decades [97].

This projected increase is driven by three factors: demographic change, ongoing transmission of resistant pathogens, and persistent gaps in access to timely diagnostics and effective antimicrobial therapy [97].

Concurrently, infectious diseases persist in their role as significant contributors to the global burden of disease, as measured by disability-adjusted life years (DALYs) [98,99].

The ongoing global pandemic has further compounded this challenge, giving rise to both direct excess mortality and indirect effects, including the disruption of essential services and routine immunization programs [100].

#### 3.2.2. Pandemics as AMR Catalysts

Pandemics can accelerate AMR through reinforcing mechanisms, including shifts toward empiric therapy, strained infection prevention and control, and disrupted surveillance [96].

During the period of the ongoing global pandemic of the SARS-CoV-2, a high level of empirical antibiotic prescribing was reported despite relatively low rates of confirmed bacterial coinfection. This phenomenon has been shown to increase selective pressure for resistant organisms [100].

Furthermore, the redirection of laboratory capacity and surveillance resources has led to a diminution in the efficacy of routine AMR monitoring in numerous settings [97].

The aforementioned effects manifested most distinctly in LMICs, where constrained laboratory capacity, inadequate stewardship infrastructure, and disrupted supply chains impeded appropriate prescribing and timely detection of resistant outbreaks [97].

#### 3.2.3. Strain on Healthcare Systems

Research has demonstrated a correlation between AMR and pandemics, indicating a collective increase in demand for intensive care, prolonged hospitalization, advanced diagnostics, and costly second-line or last-resort therapies. This phenomenon has been shown to exacerbate the pressure on health systems that are constrained by limited resources [1].

The presence of drug-resistant infections has been demonstrated to be associated with elevated rates of treatment failure and augmented healthcare expenditures, resulting in the displacement of capacity for routine, preventive, and elective services [1].

According to the findings of health system appraisals grounded in the conceptual framework of the WHO building blocks, the repercussions of the SARS-CoV-2 pandemic have had a deleterious effect on governance, financing, workforce capacity, service delivery, access to essential medicines, and health information systems with relevance to AMR response [101].

#### 3.2.4. Public Health, Societal, and Policy Implications

The convergence of pandemics and accelerating AMR poses a significant threat, as it may lead to the normalization of an era in which common infections, routine surgical procedures, obstetric care, and cancer chemotherapy become substantially more hazardous due to the diminishing effectiveness of empirical and targeted antimicrobial therapy [1].

The societal ramifications of this phenomenon are multifaceted. Productivity losses due to premature mortality and long-term disability are evident, as are widening inequities and macroeconomic drag driven by rising healthcare expenditures and reduced labor force participation [1].

Vaccination is a critical yet underutilized intervention at the intersection of pandemic preparedness and AMR mitigation [102].

Vaccines have been demonstrated to play a pivotal role in mitigating bacterial and viral infections by curtailing antimicrobial consumption and the subsequent selection pressure for resistance. This, in turn, serves to fortify the resilience of healthcare systems during periods of epidemic shocks [102].

A more comprehensive approach to AMR control necessitates a One Health strategy, acknowledging the interconnected roles of human health, animal health, agriculture, and the environment in the emergence and propagation of resistance [103,104].

Integrating AMR mitigation into pandemic preparedness frameworks, rather than treating it as a parallel agenda, will be essential for safeguarding modern medicine and building resilient, equitable health systems capable of withstanding future global health crises [98].

### 3.3. Research and Diagnostic Implications

The converging threats of pandemics and AMR underscore an urgent need for advances in diagnostics, surveillance, and early warning systems capable of keeping pace with rapidly evolving pathogens and resistance determinants [96].

Strengthened diagnostic capacity is imperative for effective patient management and the implementation of antimicrobial stewardship, real-time AMR surveillance, and pandemic preparedness at national and global levels [96].

#### 3.3.1. Need for Improved Diagnostics

Conventional culture-based diagnostics often require 24–72 h to definitively identify pathogens and determine their susceptibility, thereby delaying the initiation of targeted therapy and prolonging the use of broad-spectrum antimicrobials, which in turn may promote resistance [104]. However, even when microbiological results are available quickly, it is often difficult to interpret them clinically due to uncertainty regarding disease evolution and the distinction between colonization and active infection. This highlights the need for diagnostic approaches that are informed by the host response and aware of the context [105].

The utilization of rapid diagnostic tests (RDTs), particularly in conjunction with antimicrobial stewardship programs, has been demonstrated to enhance clinical outcomes in cases of bloodstream infection. This enhancement is evidenced by a reduction in the time to initiate optimal therapy, and, in certain contexts, a decline in mortality rates [104,106].

Molecular diagnostic approaches, including multiplex PCR panels and other rapid platforms, have been developed to support the identification of organisms from positive blood cultures and selected specimen types. However, their impact on the time taken to make actionable clinical decisions is highly context-dependent, often being constrained by the need for prior culture enrichment and challenges in clinical interpretation [107]. Metagenomic approaches offer pathogen-agnostic detection, which can serve as a valuable complement to conventional testing when standard diagnostics yield negative or slow results [108].

Plasma microbial cell-free DNA sequencing has also been clinically validated for the detection of selected AMR genetic markers in important pathogens. This finding supports earlier resistance-informed decision-making in appropriate contexts [108].

However, implementation remains constrained by costs, infrastructure requirements, and inequitable access, especially in LMICs [107].

Point-of-care diagnostics (POCT) serve to complement centralized testing, particularly within emergency departments, primary care settings, and resource-constrained environments [109].

Biomarker-guided approaches, including procalcitonin-guided algorithms in defined clinical scenarios, have the potential to reduce unnecessary antimicrobial exposure when embedded in clinical decision pathways [110].

In a similar vein, stewardship-oriented rapid testing strategies in outpatient and primary care pathways have been shown to reduce inappropriate prescribing when paired with appropriate clinical workflows [111].

#### 3.3.2. Diagnostic Stewardship, AMR and Virulence Surveillance

Optimizing the impact of diagnostic tests necessitates the implementation of diagnostic stewardship, which can be defined as a series of coordinated interventions aimed at promoting the appropriate utilization of diagnostic tests. The primary objective of these interventions is to improve patient outcomes while minimizing unintended consequences such as overtesting and misinterpreting test results [112]. In light of the increasing complexity of diagnostic pathways, clinical decision support tools incorporating data-driven and artificial intelligence (AI)-based approaches could facilitate appropriate test ordering and interpretation in certain situations. Diagnostic stewardship is inextricably linked to antimicrobial stewardship and surveillance, forming a continuum that connects testing decisions, treatment choices, and population-level resistance monitoring [107,112].

Robust AMR surveillance systems that integrate microbiological data with clinical metadata and antimicrobial consumption are essential for detecting shifts in resistance patterns, identifying high-risk clones, and informing empiric guidelines and infection prevention strategies [113].

Analyses employing GLASS-reported data demonstrate the value of harmonized surveillance and the linkage of resistance with antimicrobial consumption metrics at the country level [110].

In LMICs, routinely collected microbiology data, when standardized, validated, and benchmarked, has been shown to guide local stewardship priorities and strengthen national action plans to address AMR [111].

The efficacy of surveillance systems is contingent upon the integration of interoperable laboratory information systems, the establishment of harmonized data standards, and the implementation of governance frameworks that facilitate secure and ethical data sharing across institutional and international boundaries [113].

#### 3.3.3. Early Detection of Emerging Pathogens and Resistance Threats

The COVID-19 pandemic exposed weaknesses in early detection and highlighted the value of integrated surveillance combining multiple data streams for near real-time signal detection [96].

A growing body of research has called for implementing integrated early warning systems for AMR that utilize whole-genome sequencing, predictive analytics, and longitudinal sampling to identify emerging resistance mechanisms and high-risk clones before their widespread dissemination [113,114,115].

In order to address the aforementioned challenges, it is imperative to allocate sustained investment in scalable technologies, as well as in workforce development and laboratory capacity. The objective of this investment is to facilitate the translation of complex diagnostic and genomic data into actionable public health responses [116].

A central tenet of aligning AMR control with broader pandemic preparedness and global health security agendas [96] is the collective strengthening of diagnostics, surveillance, and early warning systems through integrated, equitable, and workforce-supported approaches.

**Figure 2:**
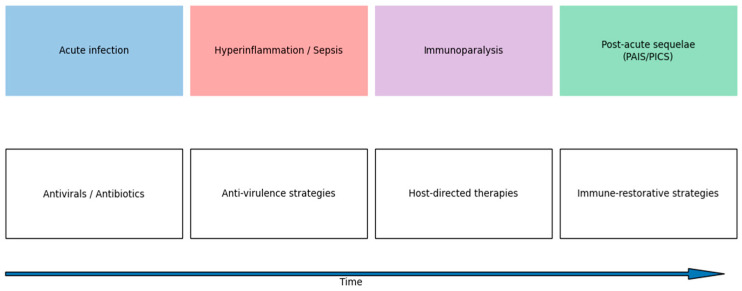
Temporal evolution of host response during severe infection. The progression of the disease is characterized by an initial phase of acute infection and pathogen burden, followed by a subsequent phase of hyperinflammation and host-mediated injury. This is then followed by a third phase of immunoparalysis and the potential development of post-acute sequelae (PACS/PICS). Therapeutic strategies are tailored to the disease stage and encompass antimicrobial therapy, anti-virulence approaches, host-directed therapies, and immune-restorative interventions.

The temporal relationship between host responses and stage-specific therapeutic opportunities is illustrated in Figure 2.

## 4. Therapeutic Targets and Strategies

Therapeutic strategies for infectious diseases can be broadly divided into two categories: direct pathogen-targeted interventions and approaches that interfere with virulence mechanisms or modulate host immune responses [28,117]. While classical antimicrobial drugs remain central to treatment, their effectiveness is compromised not only by AMR but also by biofilm-associated growth, toxin-driven disease, and host-related factors, underscoring the need for adjunctive and host-directed therapeutic [80,83]. Beyond their antimicrobial activity, several antibacterial and antifungal agents exert immunomodulatory effects on the host, which may influence disease progression and recovery and represent an important complementary area for future investigation. This necessitates the development of complementary and combination-based therapeutic concepts [118].

### 4.1. Direct Pathogen-Targeted Therapies

Direct pathogen-targeted therapies are designed to inhibit microbial components that are essential for replication or survival. In the field of antiviral treatment, significant advancements have been made in the development of highly effective agents directed against conserved viral enzymes. These enzymes include proteases and RNA-dependent RNA polymerases, as well as structural proteins that play a crucial role in the entry and assembly of viruses [69,119,120]. A notable example is the SARS-CoV-2 main protease (Mpro), a conserved cysteine protease that is essential for viral polyprotein processing. Structure-based drug design has facilitated the development of covalent Mpro inhibitors, including nirmatrelvir (PF-07321332), which impedes viral replication and maintains activity across multiple coronavirus variants due to the robust conservation of the catalytic pocket [121,122,123].

Polymerase-directed antiviral therapy is exemplified by remdesivir, a nucleotide analogue that targets the viral RNA-dependent RNA polymerase. In hospitalized patients with confirmed cases of SARS caused by the SARS-CoV-2, remdesivir has been demonstrated to significantly reduce recovery time when compared to a placebo. This finding serves to substantiate the hypothesis that the treatment of severe viral infections can be effectively targeted by means of polymerase-directed antiviral therapy [72].

Classical antibiotics function by inhibiting several crucial processes within bacterial cells, including cell wall and protein synthesis, nucleic acid metabolism, and key enzymatic pathways. However, the clinical utility of these agents is increasingly constrained by resistance mechanisms such as enzymatic drug inactivation, target modification, the expression of efflux pumps and reduced membrane permeability [83]. Despite their indispensable role, antibiotics are often unable to adequately address host-mediated immunopathology, contributing to prolonged hospitalization, tissue damage and adverse clinical outcomes, even when microbiological control is achieved. This situation highlights the need for additional and alternative therapeutic strategies [28].

### 4.2. Virulence-Directed Strategies as Next-Generation Antimicrobials

Virulence-directed strategies, frequently termed NGAs, endeavor to attenuate pathogenicity as opposed to directly eradicating the microorganism. The targets of these regulatory networks include quorum sensing (QS), biofilm formation, adhesion, toxin production, and global regulatory networks controlling virulence gene expression [77,117,124]. These processes are typically non-essential for microbial viability; therefore, their inhibition may exert less selective pressure for resistance compared with bactericidal antibiotics [77,124].

Virulence inhibition has been demonstrated to reduce tissue damage and facilitate immune-mediated clearance, particularly in cases of chronic or biofilm-associated infections. Consequently, combination approaches integrating virulence inhibitors with conventional antibiotics are considered attractive, as QS blockade or biofilm disruption can restore antibiotic susceptibility and reduce resistance-associated treatment failure [77,80,81,124].

### 4.3. Bacteriophage Therapy

Bacteriophage therapy utilizes lytic phages as biologically specific antimicrobials, capable of selectively infecting and lysing bacterial pathogens, including multidrug-resistant strains that are refractory to antibiotic treatment [125,126,127]. Contemporary clinical applications generally entail personalized or cocktail-based phage preparations targeting pathogens such as *Pseudomonas aeruginosa* or *Acinetobacter baumannii* in complex soft tissue, osteoarticular, or respiratory infections, with administration via topical, parenteral, or inhalational routes [125,126,127,128].

A recent multicenter, multinational, retrospective study of 100 consecutive personalized phage therapy cases reported clinical improvement in 77.2% of patients and microbiological eradication in 61.3% of targeted infections. Concomitant antibiotic therapy has been demonstrated to be associated with improved outcomes, underscoring the significance of phage–antibiotic synergy [128].

The regulatory challenges associated with this process encompass quality control, standardized susceptibility testing, and product classification. In the United States, the FDA has implemented pathways that include cleared Investigational New Drug (IND) and Expanded Access IND programs for specific phage-bank products, reflecting an evolving regulatory landscape [127,129]. In Europe, the European Pharmacopoeia adopted the general chapter Phage therapy medicinal products (5.31) in 2024, with implementation entering into force in 2025, providing harmonized quality standards for phage-based medicinal products [130].

### 4.4. Host-Directed Therapies and Immunomodulation

The objective of host-directed therapies (HDTs) is to enhance antimicrobial effector mechanisms or to limit detrimental immunopathology. Key targets of this research include inflammasome activation, STING signaling, immunometabolic pathways, and autophagy, all of which influence intracellular pathogen clearance and inflammatory tissue damage [28].

The clinical efficacy of immunomodulation in the treatment of severe viral pneumonia has been well-documented. In the context of SARS-CoV-2, the phenomenon of hyperinflammation has been observed to predominate over uncontrolled viral replication as a primary driver of morbidity and mortality. Accordingly, glucocorticoids and targeted immunomodulators—including IL-6 receptor antagonists and JAK inhibitors—have demonstrated efficacy in specific patient populations [74,131]. In the RECOVERY trial, the administration of tocilizumab in conjunction with standard corticosteroid therapy resulted in a reduction in 28-day mortality among hospitalized patients suffering from hypoxia and systemic inflammation (31% vs. 35%; rate ratio 0.85, 95% CI 0.76–0.94) [131]. However, it is imperative to meticulously evaluate the potential risks associated with this approach, including the possibility of secondary infections and viral reactivation, as evidenced by studies such as [90,96].

### 4.5. Monoclonal Antibodies for Infectious Diseases

Monoclonal antibodies (mAbs) are well-established therapeutic agents employed in the treatment of infectious diseases. These antibodies are utilized for a variety of purposes, including the neutralization of pathogens, toxins, and passive immunization in high-risk individuals [132,133]. Clinically approved examples include the neutralization of antibodies against respiratory viruses, such as RSV and SARS-CoV-2, as well as toxins-neutralizing antibodies that reduce the recurrence risk of *Clostridioides difficile* infections without disrupting the commensal microbiota [132,133].

Advancements in antibody engineering have led to the development of next-generation mAbs that exhibit enhanced pharmacodynamics and an extended half-life. These mAbs also demonstrate improved Fc-mediated effector functions and the capacity to exist in bispecific or multispecific formats. These agents are increasingly explored in combination with antiviral or immunomodulatory therapies, facilitated by technological advances in human immunoglobulin transgenic models and single-cell B-cell receptor sequencing [133,134].

### 4.6. CAR-Based Immunotherapy

Chimeric antigen receptor (CAR)-based immunotherapy is a therapeutic modality that adapts principles from oncology to infectious diseases. This approach involves engineering immune cells to recognize pathogen-derived or infection-associated antigens independently of HLA presentation [135,136,137,138]. Second-generation CAR constructs combine an extracellular antigen-binding domain with CD3ζ and co-stimulatory domains (CD28 or 4-1BB), thereby enabling robust activation, proliferation, and persistence of engineered cells [139,140,141].

In the context of infectious disease, CAR-based approaches necessitate meticulous consideration of safety concerns, as uncontrolled activation has the potential to intensify inflammation or result in on-target off-tissue toxicity [142,143]. Infection-adapted CAR designs, therefore, emphasize controllability through suicide switches, transient expression strategies, or logic-gated activation [139,140,141,142,143]. The extant evidence base for CAR immunotherapy in viral infections is summarized in Table 4.

## 5. Design and Development of New Therapeutics

### 5.1. Computational and Experimental Discovery Pipelines

The identification of novel anti-infective agents is increasingly dependent on integrated computational and experimental pipelines. These pipelines facilitate the efficient exploration of chemical space, rapid hypothesis testing, and the early prioritization of candidates with translational potential [155,156,157]. Structure- and ligand-based computational methods, including molecular docking, pharmacophore modeling, and quantitative structure–activity relationships (QSAR), constitute core components of contemporary small-molecule discovery. These methods have been extensively applied to viral and bacterial targets relevant to emerging infections and AMR [155,158]. By facilitating the virtual screening of extensive compound libraries and the in silico optimization of affinity and selectivity, these methodologies effectively mitigate experimental attrition and expedite the transition from hit identification to lead optimization [155,156,157,158].

Machine-learning (ML) and deep-learning approaches further extend these strategies by leveraging large chemical, biological, and omics datasets to predict bioactivity, prioritize chemotypes, identify repurposing opportunities, and support the early assessment of absorption, distribution, metabolism, excretion, and toxicity (ADMET) properties [159,160,161]. A seminal example is the ML-driven identification of halicin, a structurally distinct and previously under-explored chemotype with broad-spectrum antibacterial activity, including activity against multiple priority pathogens, such as multidrug-resistant Gram-negative bacteria and *Mycobacterium tuberculosis* [162]. This and subsequent studies illustrate how machine learning (ML)-enabled pipelines can expand accessible chemical space and accelerate hit identification and prioritization during AMR crises and outbreaks of emerging infectious diseases [156,163].

The efficacy of computational methods is maximized when integrated within iterative in silico–in vitro discovery cycles. In these cycles, predicted hits undergo rapid evaluation in biochemical and cell-based assays, and experimental data is utilized to refine subsequent computational predictions [155,162]. Such integrated pipelines facilitate the dynamic reallocation of candidates as resistance patterns, epidemiological conditions, or target product profiles evolve, and are particularly advantageous for outbreak-responsive discovery against WHO-priority pathogens [157,158].

In addition to single-target optimization, systems biology and network-based analyses are increasingly being used to complement structure-based and ML-based approaches. These complementary methods involve the modeling of host–pathogen interactions, pathway redundancy, and polypharmacology [164,165]. These methods facilitate the identification of multi-target and host-directed strategies that may exhibit enhanced resilience to resistance development and demonstrate greater alignment with complex disease biology. Consequently, these strategies serve to fortify the connection between discovery-stage hypotheses and clinically meaningful intervention strategies [164,165]. Concurrently, advancements in computational design are being expanded beyond small molecules to biologics and emerging therapeutic modalities, including monoclonal antibodies, bispecific constructs, and nucleic acid-based interventions. This diversification of the scope of discovery platforms applicable to infectious diseases is a significant development in the field [165,166].

The integration of developability considerations at the outset and in an explicit manner—including pharmacokinetic/pharmacodynamic (PK/PD)-relevant exposure targets, safety margins, formulation constraints, and translational feasibility—is increasingly recognized as essential for efficient progression from computational hits to clinically viable candidates [159,160,161]. The integration under consideration provides a natural interface between discovery pipelines and translational pharmacology frameworks. This ensures that prioritized candidates are aligned with downstream regulatory and clinical requirements.

It is evident that these computational and experimental strategies form a spectrum of complementary discovery platforms. As illustrated in Table 5, a compendium of the prevailing computational and integrated methodologies employed in contemporary therapeutic discovery has been assembled, with an emphasis on their fundamental functions, their pertinence to the domain of infectious diseases, and the preponderance of supporting evidence.

### 5.2. Translational Pharmacology and Regulatory Frameworks

Translational pharmacology is the field that quantitatively links candidate therapeutics emerging from discovery pipelines to clinically effective regimens. This linkage is achieved through the integration of PK/PD, and disease biology [159,160]. The formalization of exposure-response relationships at both the individual and population levels is a critical component of translational PK/PD frameworks. These frameworks facilitate the rational selection of doses, the optimization of schedules, and the monitoring of treatments across diverse patient populations [168]. The paradigm of model-informed drug development (MIDD) for anti-infectives is predicated on the integration of pathogen characteristics, host factors, immune responses, and resistance dynamics into mechanistic or semi-mechanistic models. These models function as a nexus, thereby facilitating the convergence of preclinical findings with clinical decision-making [160,163].

The application of population PK/PD models and model-based meta-analyses has proven instrumental in the optimization of antiretroviral therapy. These models offer a valuable illustration of the quantitative translation process from discovery to clinical practice. The ENCORE1 trial demonstrated that a daily dosage of 400 mg of efavirenz is non-inferior to the standard 600 mg dosage while concomitantly reducing adverse events. This result underscores the efficacy of exposure-response analysis, when supported by appropriate trial design, in enabling dose optimization without compromising efficacy [169]. Complementary model-based meta-analyses and within-host viral dynamics models provide further insight into the design of combination therapies, adherence strategies, and resistance-suppressive exposure targets in the context of HIV infection [170,171].

In patients with critical illnesses, pathophysiological alterations and variable pathogen susceptibility pose challenges to fixed-dose antibiotic regimens, thereby highlighting the limitations of one-size-fits-all dosing strategies. Substantial exposure variability is attributable to changes in renal clearance, organ dysfunction, and volume of distribution, particularly in the case of time- and concentration-dependent antibiotics. The optimization of outcomes and the limitation of resistance in severe bacterial infections and sepsis is contingent upon individualized dosing approaches guided by PK/PD targets, such as fT > MIC or AUC/MIC. These approaches are supported, where feasible, by therapeutic drug monitoring [168].

In circumstances where the utilization of conventional human efficacy trials is either impracticable or ethically questionable, particularly in the context of high-lethality or rare emerging infections, the US Food and Drug Administration’s Animal Rule emerges as a regulatory framework. This framework facilitates the demonstration of efficacy in predictive animal models, with supporting data encompassing human PK, safety, and other relevant parameters serving to substantiate clinical dosing regimens [172]. The following elements are imperative for the successful execution of this endeavor: the selection of pertinent animal species, the establishment of clinically meaningful endpoints, and the substantiation that human exposures align with or surpass efficacious animal exposures. This ensures a direct correlation between preclinical efficacy and translational pharmacology principles, thereby facilitating the advancement of pharmaceutical products from the laboratory to the clinical setting [172].

Real-world evidence (RWE) and observational data have become increasingly important in complementing clinical trials and modeling approaches, particularly during public health emergencies. When interpreted in conjunction with mechanistic understanding and controlled trial data, RWE has the potential to facilitate post-authorization dose optimization, safety evaluation, and effectiveness assessment, thereby completing the cycle between initial discovery, clinical deployment, and ongoing learning [173].

Adaptive platform trials represent a further cornerstone of translational and regulatory strategy for emerging infections. Master-protocol designs facilitate concurrent evaluation of multiple interventions using response-adaptive randomization and flexible modification of treatment arms, enhancing efficiency, ethical balance, and generalizability during pandemics and other rapidly evolving outbreaks, while maintaining compatibility with model-informed and exposure–response-based decision-making [174].

The integration of translational pharmacology and regulatory frameworks facilitates the effective transition of preclinical candidates to clinically actionable interventions. As illustrated in Table 6, a comprehensive overview of pivotal translational platforms pertinent to the development of novel infection therapeutics is provided. This overview encompasses their designated roles in the therapeutic development process and the primary types of supporting evidence available.

## 6. Discussion

This review proposes an integrated conceptual framework in which infectious disease therapeutics are situated along a continuum ranging from pathogen-directed and virucidal strategies to host-directed and immune-based interventions, embedded within quantitative, translational, and implementation-oriented structures. By explicitly integrating mechanistic insights into host–pathogen interactions with clinical phenotypes, pharmacology, and health-system constraints, this framework aims to support rational therapeutic selection, optimization, and combination across endemic infections and future pandemic threats [28].

### 6.1. Integration of Host–Pathogen Mechanisms with Clinical Implications

The severity of disease and the clinical outcomes observed in cases of bacterial and viral infections are the result of a dynamic interplay between pathogen-related factors and host responses. These pathogen-related factors include microbial burden, virulence determinants, and resistance mechanisms. The host responses encompass innate and adaptive immunity, immunopathology, and subsequent immune suppression [28,175]. A mounting body of evidence suggests that dysregulated host responses, characterized by excessive innate immune activation, inflammasome signaling, cytokine storm, and sepsis-associated immunoparalysis, frequently become the predominant drivers of organ dysfunction, post-acute sequelae, and long-term disability. This phenomenon contrasts with the notion that these outcomes are merely passive consequences of pathogen replication alone [175,176]. These observations call into question the conventional therapeutic paradigms that focus exclusively on pathogens. Instead, they support the development of stage-adapted strategies that involve the timely management of pathogens in conjunction with the context-specific modulation of host responses.

This heterogeneity reflects pathogen factors, disease stage, and baseline modifiers, such as age-related immune remodeling and immunosuppression. These factors reinforce the limitations of uniform, one-size-fits-all therapeutic strategies and underscore the need for context-aware immune monitoring and stage-adapted interventions.

From a clinical perspective, this integration necessitates the mapping of molecular and cellular mechanisms onto recognizable syndromes, including but not limited to severe pneumonia, acute respiratory distress syndrome, sepsis, and post-acute infection syndromes. Biomarker-guided approaches that are capable of capturing both pathogen dynamics and host-response phenotypes—including inflammatory mediators, tissue injury markers, and immune functional signatures—are therefore central to patient stratification, trial enrichment, and therapeutic decision-making [177,178]. It is imperative to acknowledge the substantial inter-individual and temporal heterogeneity that characterizes this condition. This heterogeneity implies that interventions targeting identical pathways may yield benefits within defined biological windows while causing harm outside them. This underscores the necessity for iterative refinement of mechanistic-clinical alignment [91,177].

The clinical translation of host- and virulence-directed strategies is contingent on the timely and context-appropriate implementation of diagnostic procedures. The ability to rapidly identify pathogens, assess resistance profiles, and characterize host responses is paramount for the effective implementation of stage-adapted therapies and the avoidance of indiscriminate immunomodulation [179,180]. In the absence of integration of diagnostics into therapeutic pathways, the conceptual advantages of host-directed and virulence-targeted interventions risk remaining theoretical rather than actionable, particularly in acute care and resource-limited settings [179,180].

### 6.2. Comparative Analysis of Pathogen-, Virulence-, Host- and Immune-Based Therapies

Direct pathogen-targeted agents remain a cornerstone of acute infectious disease management, particularly in cases where expeditious pathogen reduction is imperative to avert irreversible tissue damage and onward transmission. Antiviral agents that target conserved viral proteases and polymerases, in conjunction with antibiotics that are directed against essential bacterial processes, have resulted in substantial improvements in patient outcomes. However, these treatments are hindered by the emergence of resistance, limited spectra, biofilm-associated tolerance, and development timelines that lag behind epidemiological change [181]. The optimal clinical utilization of these agents is increasingly contingent upon the integration of PK/PD-informed dosing methodologies, rational treatment shortening strategies, and combination treatment approaches designed to both suppress resistance and preserve the integrity of the host and microbiome [182].

A conceptual shift has been observed in the field, moving from a focus on pathogen eradication to a new approach centered on functional disarmament. This shift is characterized by the targeting of quorum sensing, biofilm formation, adhesins, toxins, and global regulatory systems. It is hypothesized that these approaches interfere with pathogenicity rather than viability. This is believed to result in a reduced selective pressure for resistance and to restore or potentiate the activity of conventional antimicrobials, particularly in chronic and device-associated infections [183]. Nonetheless, translational challenges persist, encompassing the demonstration of in vivo target engagement, the delineation of clinically meaningful endpoints, and the mitigation of risks associated with biofilm disruption and pathogen dissemination.

Host-directed therapies and immune-based interventions form a complementary axis focused on modulating host pathways such as pattern-recognition receptor signaling, inflammasome activation, interferon responses, immunometabolism, autophagy, and lymphocyte function [28,175,176]. The field has seen significant advancements in recent years, with the development of monoclonal antibodies and emerging engineered immune modalities. These innovations have led to a proliferation of new treatments, offering targeted effector functions. However, these advances have also introduced novel safety concerns, including the potential for cytokine release and the exacerbation of immune dysregulation [176].

It is important to note that the limited success of many host-directed and immunomodulatory interventions in sepsis and severe infection has been partly attributed to inadequate consideration of host-response heterogeneity and disease stage in trial design [91,177]. Conventional “one-size-fits-all” approaches are likely to result in the dilution of treatment effects when applied across biologically diverse patient populations. These observations underscore the necessity for biomarker-informed enrichment strategies, adaptive trial designs, and dynamic treatment algorithms that align therapeutic mechanisms with evolving host–pathogen states [91,137,177,184].

When considered as a whole, these approaches should not be regarded as competing alternatives but rather as components of rational, mechanism-informed combinations tailored to pathogen characteristics, host-response phenotypes, disease stage, and health-system capacity.

### 6.3. Cross-Cutting Challenges: Resistance, Toxicity, Cost, Regulation, and Access

Across therapeutic classes, antimicrobial resistance, toxicity, and implementation constraints converge as major barriers to sustainable impact [181,182]. Notwithstanding the substantial global burden, the development of new antibacterial agents remains limited. Many of these agents offer only incremental progress in the fight against priority pathogens, rather than revolutionary advances [118]. Concurrently, host-directed, virulence-directed, and immune-based therapies frequently depend on complex biologics or advanced manufacturing platforms that are challenging to implement on a large scale, especially in low-resource environments [28,185].

The regulatory complexity further influences the translational trajectories of these cells. The advent of novel therapeutic modalities, such as bacteriophage therapy, has given rise to the necessity for the development of bespoke quality, safety, and regulatory pathways. These novel pathways must diverge from those currently employed in the context of conventional small-molecule antimicrobials [183,185]. In the context of high-consequence emerging infections, where the execution of human efficacy trials is impractical, there is a compelling rationale for placing reliance on structured translational frameworks. These frameworks underscore the critical importance of predictive preclinical models and robust PK/PD strategies. Such strategies are instrumental in facilitating a bridge between the experimental efficacy of a given intervention and its human dosage [172,182].

In addition to regulatory approval, the successful deployment of novel anti-infective strategies is contingent upon the implementation of scientific frameworks that address workforce training, diagnostic–therapeutic integration, supply chains, and antimicrobial stewardship infrastructure [7,14]. Even highly effective interventions may fail to deliver population-level benefit if they cannot be embedded into real-world clinical workflows, particularly during periods of health-system stress such as pandemics. On a global scale, elevated acquisition expenses, disorganized intellectual property arrangements, and constrained manufacturing capacity pose a persistent threat to equitable access, particularly in low- and middle-income countries [185,186].

The central role of AI, ML, PK/PD, and translational frameworks in preparedness is well-established.

The role of AI and machine learning in anti-infective discovery and development is indisputable. These technologies enable efficient exploration of chemical space, prediction of bioactivity and ADMET properties, and identification of novel antimicrobial candidates [155,156,167]. ML-driven discovery pipelines have demonstrated the feasibility of mining large-scale biological and microbiome data to generate diverse candidate sets, offering potential acceleration pathways during antimicrobial resistance crises and emerging outbreaks [162,187].

Translational pharmacology and PK/PD modeling provide the quantitative foundation that links discovery to clinical implementation, enabling rational dose optimization, combination design, and adaptive evidence generation during public health emergencies [159,160,161,182]. The incorporation of PK/PD considerations at the outset of therapeutic development facilitates the identification of candidates that are biologically promising, manufacturable, affordable, and scalable across a range of health-system contexts [160,168].

Consequently, future pandemic preparedness will be contingent not on a singular therapeutic class but rather on the integration of host–pathogen mechanistic insight, rapid diagnostics, quantitative PK/PD frameworks, and equitable implementation into coherent, adaptive response platforms. Such integration is imperative for translating scientific advances into durable global resilience against both antimicrobial resistance and emerging infectious threats.

## 7. Conclusions and Future Directions

The evolving therapeutic paradigm in the domain of infectious diseases is characterized by the convergence of precision immunomodulation, diversified anti-infective platforms, and data-driven translational frameworks. This review emphasizes the importance of integrating a mechanistic understanding of host–pathogen interactions with emerging therapeutic and translational approaches to improve outcomes in severe infections, sepsis, and future pandemics. In light of the persistent challenges posed by sepsis, the accelerating emergence of antimicrobial resistance, and the recurrent nature of pandemics, the prospect of future progress is contingent upon the implementation of enhanced mechanistic specificity in the targeting of host–pathogen interactions. This approach must be accompanied by the assurance that innovations are scalable, affordable, and globally implementable across a range of health-system contexts. Although vaccination remains a cornerstone of infectious disease prevention, the present review has focused on therapeutic strategies that address established and severe infections.

### 7.1. Precision Targeting of Innate Immunity and Host Responses

Innate immune sensors, inflammasomes, and complement and downstream cytokine networks represent promising yet highly context-sensitive targets for precision intervention in severe infection and sepsis [50,85,188,189]. Evidence from sepsis and related syndromes demonstrates that excessive activation of pattern-recognition receptor pathways, inflammasome signaling, and cytokine cascades contributes to acute respiratory distress syndrome, multiorgan failure, and post-acute morbidity. Later disease phases are frequently characterized by immunoparalysis and heightened susceptibility to secondary infection [87,89,188,189]. Therapeutic strategies that precisely modulate innate pathways—such as targeting the NLRP3 inflammasome, cGAS–STING signaling, complement activation, or JAK–STAT pathways—on the basis of immune phenotyping, biomarker profiles, and dynamic clinical criteria may therefore offer improved outcomes compared with uniform immunosuppression [90,189,190].

The incorporation of such precision immunomodulation into adaptive platform trials and real-time biomarker-guided treatment algorithms provides a pragmatic approach to balancing pathogen control with the mitigation of immunopathology, while potentially reducing long-term disability following severe infection and sepsis.

### 7.2. Expansion of NGAs, Phage Therapy, and Antibody Platforms

NGAs that attenuate virulence, bacteriophage therapy, and advanced antibody platforms are expected to play an increasingly central role in managing antimicrobial resistance while limiting further selective pressure on classical antimicrobial targets [117,191]. Virulence-directed NGAs targeting quorum sensing, biofilm formation, adhesins, toxins, and global regulatory systems expand the therapeutic toolbox by disarming pathogens rather than killing them, with particular promise in chronic, relapsing, and device-associated infections when deployed as adjuncts to antibiotics [117,191].

In parallel, growing clinical experience with personalized and cocktail-based bacteriophage therapy against multidrug-resistant bacterial pathogens demonstrates feasibility but underscores the need for standardized susceptibility testing, robust manufacturing and quality frameworks, and harmonized regulatory pathways to enable broader and more equitable clinical adoption [188,192]. Monoclonal antibodies and engineered derivatives—including Fc-extended, bispecific, and multispecific constructs—will continue to expand as platforms for pathogen and toxin neutralization, passive immunization of high-risk populations, and combination regimens with antivirals or immunomodulators, provided that challenges related to cost, scalability, and global access can be addressed.

### 7.3. Clinical Evaluation of CAR-Based Therapies in Infection

Chimeric antigen receptor (CAR)-based immunotherapy for infectious diseases remains at an early translational stage, with the majority of evidence derived from preclinical models and limited proof-of-concept studies in viral infections. Although the development of CAR technologies has been predominantly focused on the field of oncology, recent studies indicate the possibility of redirecting immune effector cells against infected targets in a manner that is independent of conventional antigen presentation pathways [144,193]. However, the inflammatory milieu characteristic of severe infections and sepsis—in conjunction with the risks of cytokine release syndrome, on-target off-tissue toxicity, and interference with endogenous immune regulation—necessitates particularly cautious clinical development.

Future directions should prioritize infection-adapted CAR designs that emphasize controllability, reversibility, and safety, including suicide switches, transient expression platforms, and logic-gated activation. It is imperative to undertake meticulously designed early-phase clinical trials in narrowly defined, high-risk indications. These trials must be supported by intensive immunomonitoring and long-term follow-up to ascertain whether CAR-based strategies can deliver enduring clinical benefit. Only then can the complexity and cost of these strategies be justified in the context of infectious disease settings.

### 7.4. Integrating Computational Design, Translational Pharmacology, and Regulatory Innovation

It is imperative to emphasize the necessity of a more profound integration of computational discovery, translational pharmacology, and adaptive regulatory frameworks. This integration is crucial for facilitating expeditious and commensurate therapeutic responses to emerging pathogens. The integration of AI and machine-learning-enabled pipelines with structure- and ligand-based design and systems biology approaches has already enabled the prioritization of small molecules, biologics, and host-directed strategies that are active against resistant and high-priority pathogens. This integration also incorporates early developability and ADMET considerations [191]. Iterative in silico–in vitro discovery cycles, supported by network-based analyses of host–pathogen interactions, offer a means to identify multi-target and host-directed interventions that are more robust to resistance and better aligned with complex disease biology.

At the translational level, the quantitative scaffolding for efficient dose selection, regimen optimization, and individualized dosing in critical illness and sepsis is provided by population pharmacokinetic/pharmacodynamic modeling, model-informed drug development, and within-host viral and bacterial dynamics models [160,167,168]. The alignment of these approaches with adaptive platform trials, the structured use of real-world evidence, and regulatory mechanisms such as the Animal Rule have the potential to reduce the time interval from preclinical efficacy to emergency deployment while maintaining the rigor of benefit–risk assessment [172,174]. In order to ensure expeditious discovery, evaluation, implementation, and equitable access to novel therapeutics directed towards pathogens, virulence, hosts, and immune systems in the event of future infectious disease threats, it is imperative to integrate computational design, quantitative pharmacology, diagnostic stewardship, and antimicrobial resistance surveillance within a unified preparedness framework [97,112].

## Figures and Tables

**Table 1:** Innate immune sensors in infectious diseases: mechanisms, implications and therapeutic relevance.

**A. Canonical Innate Immune Sensor Families**							
**Pathway/Sensor**	**Core Sensing and Signaling Mechanisms**	**Major Infectious Triggers**		**Clinical Relevance in Infection**		**Therapeutic Relevance**	**Primary Evidence**
Toll-like receptors (TLRs)	Membrane and endosomal PRRs sensing PAMPs/DAMPs; MyD88- or TRIF-dependent signaling; activation of NF-κB, MAPKs and IRFs; induction of pro-inflammatory cytokines and type I/III IFNs [11,12,29].	Bacterial LPS, lipoproteins, flagellin; viral ssRNA, dsRNA, CpG DNA [11,12,29].		Essential for early antibacterial and antiviral defence; excessive activation contributes to cytokine storm, ARDS and sepsis; MyD88/IRAK-4 deficiency predisposes to severe infections [40].		Vaccine adjuvants (TLR agonists, clinical); TLR antagonists and pathway modulators (preclinical–clinical) [38,39].	Clinical, genetic, preclinical [11,12,29,38,39,40]
C-type lectin receptors (CLRs)	Cell-surface carbohydrate recognition receptors; Syk–CARD9 signaling; NF-κB activation and Th17-skewed responses [41,42,43].	Fungi (*Candida, Aspergillus* spp.); selected mycobacteria [41,42,43].		Critical for antifungal immunity; CARD9 deficiency linked to invasive fungal disease [42].		CLR-based vaccine adjuvants and Syk/CARD9 modulation (preclinical) [43,44].	Genetic, preclinical [41,42,43]
NLRs/inflammasomes and AIM2 inflammasome	Cytosolic sensing of microbial ligands and cellular stress; inflammasome assembly (NLRP3, NLRC4, NLRP1, and AIM2); caspase-1 activation, IL-1β/IL-18 release, pyroptosis [15,34,36].	Intracellular bacteria, viruses, fungi; toxins and danger signals [15,34].		Required for pathogen clearance; dysregulated activation drives hyperinflammation, ARDS and severe infection [15,34].		NLRP3 inhibitors; IL-1/IL-18 blockade (preclinical–clinical) [36].	Preclinical, limited clinical [15,34,36]
RIG-I-like receptors (RLRs)	Cytosolic RNA helicases (RIG-I, MDA5); MAVS signaling; induction of type I/III IFNs and ISGs [45,46].	RNA viruses including influenza and coronaviruses [45,46,47].		Central antiviral sensors; inborn errors cause life-threatening viral disease; excessive IFN contributes to immunopathology [45,47].		Synthetic RLR agonists (antiviral/oncology); IFN-pathway modulation (preclinical–clinical) [45].	Genetic, preclinical, limited clinical [45,46,47]
cGAS–STING pathway	Cytosolic DNA sensing by cGAS; cGAMP–STING activation; TBK1–IRF3 and NF-κB signaling [14,48,49].	DNA viruses; intracellular bacteria; self-DNA during tissue damage [14,48,49].		Protective antimicrobial responses; chronic activation linked to interferonopathies and tissue injury [48,49].		STING agonists (vaccines, oncology); cGAS/STING inhibitors (preclinical–early clinical) [14,48,49].	Preclinical, early clinical [14,48,49].
Complement and soluble PRRs	Complement activation with collectins/pentraxins; opsonization, lysis, modulation of inflammation and coagulation [50,51].	Broad range of bacteria and viruses; prominent in sepsis and severe viral infections [51,52,53].		Deficiency increases infection risk; overactivation contributes to immunothrombosis and organ failure [51,52].		C3/C5 or C5aR inhibition (preclinical–clinical) [52].	Clinical, preclinical [50,51,52,53].
**B. Cross-Cutting Innate Immune Sensing Pathways**							
**Pathway**	**Description**		**Clinical Relevance**		**Therapeutic Relevance**		**Primary Evidence**
Crosstalk with adaptive immunity	PRR signaling in dendritic cells and B cells shapes antigen presentation, T-cell polarization and antibody responses [30,44].		Determines durability and quality of protective immunity; defects impair vaccine responses [30,44].		Rational PRR-based vaccine adjuvant design (clinical–preclinical) [30,44].		Clinical, preclinical [30,44]
Sensing of cell death and DAMPs	Detection of DAMPs (e.g., mitochondrial DNA, oxidized lipids) by inflammasomes and cGAS–STING amplifies inflammation [36,37,49].		Secondary driver of tissue injury during severe infection [37].		Targeting DAMP–PRR axes to limit immunopathology (preclinical) [37].		Preclinical [36,37,49]

Evidence categories are defined as follows: Preclinical: Evidence derived from in vitro systems, organoids, or animal models. Genetic: Evidence from human inborn errors of immunity or robust genetic associations. Structural: High-resolution molecular structures substantiating the proposed mechanism. Early clinical: First-in-human or small clinical studies that do not provide definitive evidence of efficacy. Clinical: Evidence from controlled clinical studies demonstrating clinical relevance or efficacy. Abbreviations: PRR, pattern recognition receptor; PAMP, pathogen-associated molecular pattern; DAMP, damage-associated molecular pattern; TLR, Toll-like receptor; CLR, C-type lectin receptor; NLR, NOD-like receptor; RLR, RIG-I-like receptor; IFN, interferon; IRF, interferon regulatory factor; NF-κB, nuclear factor κB; MAPK, mitogen-activated protein kinase; ARDS, acute respiratory distress syndrome.

**Table 2:** Drug targets, mechanisms and therapeutics against SARS-CoV-2.

**A. Viral and Host Targets**					
**Viral/Host Target**	**Mechanistic Role**	**Clinical/Pathophysiological Implications**	**Representative Therapeutics/Strategies**	**Key Clinical Outcomes**	**Primary Evidence**
Spike (S)—RBD–ACE2 interaction	RBD–ACE2 binding mediates attachment and initiates entry; prefusion S is the dominant neutralization target [54,55,56].	Key determinant of transmissibility and tissue tropism; central antigen for antibody- and vaccine-mediated protection [54,55].	Neutralizing monoclonal antibodies [65]; vaccines targeting prefusion S [66,67].	Vaccination reduces risk of symptomatic infection, severe disease, and death in randomized trials and real-world studies; many monoclonal antibodies have lost neutralizing activity against emerging variants [54,55,56,65,66,67].	Structural, clinical [54,55,56,65,66,67]
Host proteases (TMPRSS2; endosomal cathepsins)	Host protease priming at S1/S2 and S2′ enables plasma membrane fusion and/or endosomal entry [56,57].	Cell-type-dependent entry route affects susceptibility to entry inhibitors [56,57].	TMPRSS2 inhibition (camostat blocks entry in vitro) [56].	TMPRSS2 inhibitors have not demonstrated consistent clinical benefit in randomized trials to date [56,57].	Preclinical [56,57]
Soluble ACE2 (host-directed)	Recombinant soluble ACE2 binds spike and reduces infection in engineered human tissues [68].	Candidate entry-blocking approach; translational relevance under evaluation [68].	Clinical-grade soluble human ACE2 (preclinical/early translational) [68].	Early-phase clinical studies are ongoing; definitive clinical efficacy has not yet been established [68].	Preclinical, early clinical [68]
Main protease (Mpro/3CLpro)	Cleaves pp1a/pp1ab to generate nsps required for RTC formation and replication [59].	Essential viral enzyme; conserved active site supports selective antiviral targeting [59,69].	Structure-guided Mpro inhibitors [69]; nirmatrelvir/ritonavir supported by structural studies and clinical efficacy data [70,71].	Nirmatrelvir/ritonavir reduces hospitalization and death in high-risk outpatients with early COVID-19 in randomized trials [59,69,70,71].	Structural, clinical [59,69,70,71]
Papain-like protease (PLpro)	Polyprotein processing and deubiquitination/deISGylation; antagonizes innate antiviral signaling [58,60].	Promotes replication and immune evasion [58,60].	PLpro inhibitor discovery and characterization (preclinical) [60].	No clinical efficacy data are available; development remains at the preclinical stage [58,60].	Preclinical [58,60]
RNA-dependent RNA polymerase (RdRp; nsp12 + nsp7/8)	Catalyzes genome replication and transcription; structural work defines drug-binding and replication states [61,62].	Central to viral proliferation and validated antiviral target [61,62].	Remdesivir [72].	Remdesivir provides a modest reduction in time to recovery in selected hospitalized patients but has limited or no effect on overall mortality [61,62,72].	Structural, clinical [61,62,72]
Nucleocapsid (N) and assembly factors (M, E)	N packages genomic RNA; M and E coordinate virion assembly and budding [6,63].	N is abundant and immunogenic; most established utility is in diagnostics and as a vaccine epitope component [63].	Diagnostic targets; vaccine epitope strategies (targeted N-directed therapeutics remain preclinical) [6].	No N-directed antiviral therapy has been approved; current applications are largely confined to diagnostics and experimental vaccine design [6,63].	Preclinical, diagnostic [6,63]
**B. Host Response and Supportive Strategies**					
**Pathway/Target**	**Description**	**Clinical Relevance**	**Therapeutic Implications**	**Key Clinical Outcomes**	**Primary Evidence**
Hyperinflammation (“cytokine storm” spectrum)	Dysregulated immune activation contributes to ARDS and organ failure in severe disease [31,32,33].	Major driver of severe outcomes and mortality [31,32,33].	Dexamethasone [73]; JAK inhibition in selected hospitalized patients (tofacitinib) [74].	Dexamethasone reduces mortality in patients requiring supplemental oxygen or mechanical ventilation; JAK inhibitors improve selected clinical outcomes in severe COVID-19 in randomized studies [31,32,33,73,74].	Clinical [31,32,33,73,74]
Micronutrients (vitamin D; zinc/ascorbic acid)	Adjunctive immunomodulatory hypothesis tested in randomized trials [75,76].	No clear clinical benefit demonstrated in RCTs in treatment settings [75,76].	Vitamin D3 (high-dose, hospitalized) [75]; zinc/ascorbic acid supplementation (outpatients) [76].	Randomized trials have not shown meaningful improvement in symptom duration or major clinical endpoints when used as treatment [75,76].	Clinical (negative RCTs) [75,76]

Definitions of evidence: Preclinical research involves the use of in vitro systems, organoids, or animal models to generate evidence regarding the effects of a given treatment or intervention. Structural: High-resolution molecular structures (e.g., cryo-EM or X-ray crystallography) that provide support for mechanisms or drug-target interactions. The present diagnostic evidence offers primarily support for the utility of biomarkers as a diagnostic tool, rather than for the efficacy of the therapeutic interventions in question. Early clinical: The initial clinical studies, also referred to as “first-in-human” studies, are characterized by the absence of definitive efficacy outcomes. Clinical: The efficacy of the treatment in question must be substantiated by controlled clinical studies demonstrating clinical relevance or efficacy. Clinical (negative RCTs): A meticulous examination of the randomized trials reveals an absence of substantial clinical benefit. Abbreviations: ACE2, angiotensin-converting enzyme 2; TMPRSS2, transmembrane protease serine 2; Mpro, main protease; PLpro, papain-like protease; RdRp, RNA-dependent RNA polymerase; RTC, replication-transcription complex; ARDS, acute respiratory distress syndrome. For the sake of brevity, the following table highlights a select group of representative SARS-CoV-2 antivirals, such as nirmatrelvir/ritonavir and remdesivir. It should be noted that this table does not aim to provide a comprehensive list of all approved or emerging agents, including additional Mpro inhibitors such as ensitrelvir.

**Table 3:** Next-generation antimicrobials targeting bacterial virulence: mechanisms, clinical relevance and evidence.

A. Canonical Anti-Virulence Target Classes				
Target/Pathways	Core Mechanisms	Clinical Relevance in Infections	Therapeutic Relevance	Primary Evidence
Biofilm matrix—extracellular DNA (eDNA)	Enzymatic degradation of eDNA destabilizes biofilm scaffold and enhances antibiotic and immune penetration [77,80].	Chronic lung infection (e.g., cystic fibrosis), chronic wounds, device-associated infections [77,80].	Adjunctive therapy to antibiotics; improves clearance of established biofilms. Clinical use is repurposed (mucolysis); anti-biofilm benefit is indirect. Requires caution due to dispersal-associated dissemination risk [77].	Clinical (repurposed; indirect), preclinical [77,80]
Biofilm matrix—extracellular proteins	Protease-mediated degradation of matrix proteins or antibody-mediated targeting of conserved components (e.g., DNABII family) [77,80,81].	Device-associated infections, chronic wounds, airway biofilms [80,81].	Adjunctive strategy; translational challenges include off-target proteolysis, immunogenicity, and delivery stability [77].	Preclinical [77,80,81]
Biofilm matrix—polysaccharides (e.g., PNAG)	Enzymatic degradation or inhibition of exopolysaccharide synthesis weakens biofilm cohesion [77,80].	Staphylococcal and polymicrobial biofilms, particularly on indwelling devices [80].	Adjunctive or local therapy (topical, device coatings); requires optimization of pharmacokinetics and tissue compatibility [77].	Preclinical [77,80]
Adhesins and pili/fimbriae	Inhibition of pilus assembly or adhesin–receptor interactions prevents initial attachment and colonization [77,79].	Early urinary tract, respiratory and device-associated infections [79].	Preventive or early adjunct therapy; strong rationale for prophylaxis in high-risk settings [79].	Preclinical, structural [77,79]
Quorum sensing—regulatory inhibition	Small molecules inhibit QS regulatory networks, reducing expression of toxins, adhesins and biofilm genes [77,79,81].	QS-driven infections such as *Pseudomonas aeruginosa* and *Staphylococcus aureus* [78,79].	Adjunctive anti-virulence strategy; redundancy of QS networks necessitates biomarker-guided development [81].	Preclinical, limited early clinical [77,78,79,81]
Quorum quenching—signal inactivation	Enzymatic degradation or modification of autoinducer molecules disrupts bacterial communication [77,81].	Biofilm-associated and device-related infections [77].	Adjunctive therapy; delivery, enzyme stability and in vivo target engagement remain key hurdles [81].	Preclinical [77,81]
Cyclic di-GMP signalling	Modulation of cyclic di-GMP promotes biofilm dispersal and alters motility and adhesion [77,79,81].	Chronic biofilm infections of airways, wounds and devices [77].	Adjunctive approach; careful timing and concomitant bactericidal therapy required [77,81].	Preclinical [77,79,81]
Virulence-associated two-component systems (TCSs)	Inhibition of sensor kinases or response regulators down-regulates virulence gene expression [77,79,81].	Invasive Gram-negative and Gram-positive infections [77,79].	Adjunctive strategy; selectivity and avoidance of global stress responses are critical [81].	Preclinical [77,79,81]
Toxin neutralization	Antibodies or small molecules neutralize toxins or block their delivery [78,81].	Severe toxin-mediated infections (e.g., necrotizing disease, severe pneumonia) [78].	Strong adjunct or bridge therapy; success depends on timing and antigen conservation [78].	Early clinical to clinical [78,81]

The following is a definition of the term “evidence”: Preclinical: in vitro systems, ex vivo models, or animal infection models. Structural: high-resolution molecular structures supporting mechanism. Early clinical: first-in-human or small clinical studies. Clinical: controlled clinical studies demonstrating relevance or efficacy. Clinical (repurposed): agents approved for other indications with demonstrated or plausible anti-virulence activity. Abbreviations: QS, quorum sensing; TCS, two-component system; eDNA, extracellular DNA; PNAG, poly-β-1,6-N-acetylglucosamine.

**Table 4:** CAR immunotherapy in viral infectious diseases: mechanisms and therapeutic applications.

Viral Infection/Target	Core CAR Mechanism	Clinical/Pathophysiological Implications	Therapeutic Relevance/Applications	Primary Evidence
General principles and safety	CARs combine an extracellular antigen-binding domain (typically scFv) with transmembrane and intracellular signaling modules (CD3ζ plus CD28 or 4-1BB), enabling MHC-independent recognition and activation of engineered T cells; safety engineering includes suicide switches, transient mRNA expression, and logic-gated CAR designs [139,140,141].	Viral infections and sepsis are characterized by dysregulated host immune responses; uncontrolled CAR activation may exacerbate hyperinflammation, cytokine release syndrome, or cause on-target off-tissue toxicity [142,143].	Infection-adapted CAR designs emphasize controllability, reversibility, and careful antigen selection; principles extrapolated from oncology but require infection-specific risk–benefit assessment [139,140,141,142,143].	Clinical; preclinical [139,140,141,142,143]
Epstein–Barr virus (EBV)	CAR T cells targeting EBV antigens expressed on infected B cells, most prominently latent membrane protein-1 (LMP1) or the lytic glycoprotein gp350 [137,138,144].	EBV latency and immune evasion in immunocompromised hosts predispose to EBV-associated lymphoproliferative disease; viral modulation of antigen presentation limits endogenous T-cell immunity [135,136].	LMP1- and gp350-specific CAR T cells demonstrate antigen-specific activation and killing in vitro and in vivo [137,138,144].	Preclinical; early clinical [135,136,137,138,144]
Cytomegalovirus (CMV)	CAR T cells directed against CMV glycoprotein B (gB) [145,146,147,148].	CMV reactivation causes major morbidity after transplantation [146,147].	Proof-of-concept activity demonstrated in preclinical and early clinical settings [145,148].	Preclinical; early clinical [145,146,147,148]
Hepatitis B virus (HBV)	CAR T cells recognizing hepatitis B surface antigen (HBsAg) [149,150].	Chronic HBV infection is characterized by immune tolerance and exhaustion [151,152].	Anti-HBs CAR T cells suppress HBV replication in vivo [149,150].	Preclinical; translational [149,150,151,152]
Hepatitis C virus (HCV)	CAR T cells targeting HCV envelope glycoprotein E2 [153].	Direct-acting antivirals have transformed HCV therapy and enabled global control strategies [154].	Platform demonstration for liver-directed CAR therapy [153].	Preclinical [153,154]

The following is a definition of the term “evidence”: Preclinical research encompasses in vitro systems, organoids, and animal infection models. Translational in vivo models are defined as humanized or disease-relevant in vivo platforms. The term “early clinical” refers to the initial phase of clinical studies, specifically those conducted as part of first-in-human or small clinical studies. These studies are characterized by the absence of definitive efficacy outcomes, as the investigational drugs or devices have not been sufficiently tested for their safety and effectiveness. Clinical: The term “clinical” refers to controlled clinical studies that demonstrate clinical relevance or efficacy. Abbreviations: CAR, chimeric antigen receptor; scFv, single-chain variable fragment; MHC, major histocompatibility complex; EBV, Epstein–Barr virus; CMV, cytomegalovirus; HBV, hepatitis B virus; HBsAg, hepatitis B surface antigen; HCV, hepatitis C virus.

**Table 5:** Computational and experimental approaches for therapeutic discovery.

Approach	Core Function	Relevance for Infectious Diseases	Primary Evidence
Structure- and ligand-based computational design	Prioritises and optimises candidate molecules against defined viral or bacterial targets using docking, pharmacophore modelling and QSAR before synthesis [155,157].	Accelerates identification of antiviral and antibacterial leads while reducing experimental burden for known and emerging pathogens [155,157].	Predominantly preclinical (computational and in vitro) [155,157]
AI- and ML-enabled discovery	Applies machine-learning and deep-learning models to chemical and biological datasets to predict activity, rank candidates and support early ADMET assessment [153,158,167].	Enables rapid triage of large libraries and identification of structurally distinct chemotypes active against resistant or high-priority pathogens [162,167].	Preclinical (computational, in vitro, occasionally in vivo) [156,162,167]
Integrated in silico–in vitro pipelines	Iteratively couples computational predictions with biochemical and cellular testing to refine potency, selectivity and developability [155,156].	Shortens timelines from hit identification to lead optimisation and supports outbreak-responsive discovery [155,156].	Preclinical experimental studies [155,156]
Systems- and network-based analyses	Uses systems biology and network modelling to identify multi-target and host-directed intervention strategies [164,165].	Supports development of therapeutics that may be more robust to resistance and applicable across pathogen classes [164,165].	Mechanistic and systems-level analyses [164,165]

The following is a definition of the term “evidence”: Preclinical research involves the use of in vitro systems, organoids, or animal models to generate evidence regarding the effects of a given treatment or intervention. Genetic: Evidence from human inborn errors of immunity or strong genetic associations is presented herein. Structural: The existence of evidence from high-resolution molecular structures supports the mechanism. Early clinical: The initial clinical studies, also referred to as “first-in-human” studies, are characterized by a lack of definitive efficacy. Clinical: The efficacy of the treatment in question must be substantiated by controlled clinical studies demonstrating clinical relevance or efficacy. Abbreviations: The following acronyms are used in this text: artificial intelligence (AI), machine learning (ML), quantitative structure–activity relationship (QSAR), absorption, distribution, metabolism, excretion, and toxicity (ADMET), in silico (computational modeling), in vitro (experiments performed in isolated biological systems), and in vivo (experiments performed in living organisms).

**Table 6:** Translational platforms for emerging infection therapeutics.

Platform	Role in Development	Relevance for Emerging Infections	Primary Evidence
Population PK/PD and model-informed drug development	Integrates pharmacokinetics, pharmacodynamics, pathogen characteristics and host factors to inform dose selection and study design [160,161].	Enables optimisation and individualisation of antiviral and antibiotic regimens across populations and clinical settings [160].	Clinical pharmacology and translational modelling studies [160,161]
Individualised dosing in critical illness	Uses PK/PD targets and therapeutic drug monitoring to tailor dosing in the presence of altered physiology [168].	Improves target attainment and outcomes in severe infections and sepsis [168].	Observational and interventional clinical studies [168]
Viral dynamics and resistance modelling	Describes within-host viral kinetics, treatment effects and resistance emergence to guide combination strategies [170,171].	Supports rational regimen design and resistance suppression for chronic and emerging viral infections [170,171].	Translational and clinical modelling studies [170,171]
Animal Rule-based translation	Demonstrates efficacy in predictive animal models with human PK and safety data used for dose justification [172].	Enables development of medical countermeasures for high-lethality or rare infections [172].	Animal efficacy studies with human PK/safety bridging [172]
Adaptive platform trials	Uses master protocols with response-adaptive randomisation and flexible treatment arms [174].	Increases efficiency and flexibility of therapeutic evaluation during pandemics [174].	Interventional clinical trials [174]

Evidence is defined as follows: Preclinical: Evidence derived from in vitro systems, organoids, or animal models. Genetic: There is compelling evidence derived from two sources: first, human inborn errors of immunity, and second, strong genetic associations. Structural: The existence of evidence from high-resolution molecular structures supports the mechanism. Early clinical: The initial clinical studies, also referred to as “first-in-human” studies, are characterized by a lack of definitive efficacy. Clinical: The efficacy of the treatment in question must be substantiated by controlled clinical studies demonstrating clinical relevance or efficacy. Abbreviations: Pharmacokinetics (PK) refers to the rate and mechanisms of drug absorption, distribution, metabolism, and excretion. Pharmacodynamics (PD) concern the effects of drugs on biological systems, including the modulation of physiological functions. The term “PK/PD” is used to denote the relationship between these two aspects of drug action. “AI” is an abbreviation for “artificial intelligence,” a field of study concerned with the development of computer systems capable of performing tasks that traditionally require intelligent human behavior. “In vitro” refers to experiments conducted in isolated biological systems, whereas “in vivo” refers to experiments performed in living organisms.

## Data Availability

The original contributions presented in this review are derived from the cited studies. No new data were generated or analyzed. Further inquiries can be directed to the corresponding author.

## Abbreviations

The following abbreviations are used in this manuscript:

ACE2	Angiotensin-converting enzyme 2
ADMET	Absorption, distribution, metabolism, excretion and toxicity
AIM2	Absent in melanoma 2
AMR	Antimicrobial resistance
ARDS	Acute respiratory distress syndrome
AUC	Area under the concentration–time curve
BCR	B-cell receptor
cGAMP	Cyclic GMP–AMP
cGAS	Cyclic GMP–AMP synthase
CAR	Chimeric antigen receptor
CARD9	Caspase recruitment domain family member 9
CF	Cystic fibrosis
CLR	C-type lectin receptor
CMV	Cytomegalovirus
CpG	Cytosine–phosphate–guanine (DNA motif)
CRP	C-reactive protein
DAMP	Damage-associated molecular pattern
DALY	Disability-adjusted life year
dsRNA	Double-stranded RNA
eDNA	Extracellular DNA
EBV	Epstein–Barr virus
ENCORE1	Efavirenz dose–response trial (ENCORE1)
ER	Endoplasmic reticulum
ERGIC	ER–Golgi intermediate compartment
ESBL	Extended-spectrum β-lactamase
ExoN	3′–5′ exoribonuclease (proofreading exonuclease)
fT > MIC	Fraction of time above minimum inhibitory concentration
FDA	Food and Drug Administration
Fc	Fragment crystallizable (region)
GBD	Global Burden of Disease
gB	Glycoprotein B
GLASS	Global Antimicrobial Resistance and Use Surveillance System
gp350	Glycoprotein 350
HBsAg	Hepatitis B surface antigen
HBV	Hepatitis B virus
HCV	Hepatitis C virus
HDT	Host-directed therapy
HLA	Human leukocyte antigen
HIV	Human immunodeficiency virus
ICU	Intensive care unit
IFN	Interferon
IFN-I	Type I interferon
IFN-III	Type III interferon
IND	Investigational new drug
IRAK-4	Interleukin-1 receptor-associated kinase 4
IRF	Interferon regulatory factor
ISG	Interferon-stimulated gene
ISG15	Interferon-stimulated gene 15
JAK	Janus kinase
JAK–STAT	Janus kinase–signal transducer and activator of transcription
LMIC	Low- and middle-income country/countries
LMP1	Latent membrane protein 1
LPS	Lipopolysaccharide
MAPK	Mitogen-activated protein kinase
mAb	Monoclonal antibody
MAVS	Mitochondrial antiviral signaling protein
MDA5	Melanoma differentiation-associated protein 5
MHC	Major histocompatibility complex
MIC	Minimum inhibitory concentration
MIDD	Model-informed drug development
ML	Machine learning
Mpro (3CLpro)	Main protease (3C-like protease)
MyD88	Myeloid differentiation primary response 88
N	Nucleocapsid (protein)
NGA	Next-generation antimicrobial
NF-κB	Nuclear factor κB
NLR	NOD-like receptor
NLRC4	NLR family CARD domain-containing protein 4
NLRP1	NLR family pyrin domain-containing protein 1
NLRP3	NLR family pyrin domain-containing protein 3
nsp	Non-structural protein
PAIS	Post-acute infection syndrome(s)
PAMP	Pathogen-associated molecular pattern
PASC	Post-acute sequelae of SARS-CoV-2 infection
PD	Pharmacodynamics
PICS	Post-intensive care syndrome
PK	Pharmacokinetics
PK/PD	Pharmacokinetic/pharmacodynamic
PLpro	Papain-like protease
PNAG	Poly-β-1,6-N-acetylglucosamine
POCT	Point-of-care testing
pp1a/pp1ab	Viral replicase polyproteins 1a/1ab
PRR	Pattern recognition receptor
PCR	Polymerase chain reaction
QS	Quorum sensing
QSAR	Quantitative structure–activity relationship
RBD	Receptor-binding domain
RCT	Randomized controlled trial
RdRp	RNA-dependent RNA polymerase
RECOVERY	Randomised Evaluation of COVID-19 Therapy
RIG-I	Retinoic acid-inducible gene I
RLR	RIG-I-like receptor
RWE	Real-world evidence
RSV	Respiratory syncytial virus
RTC	Replication–transcription complex
S	Spike (glycoprotein)
scFv	Single-chain variable fragment
Sepsis-3	Third International Consensus Definitions for Sepsis and Septic Shock
SOFA	Sequential Organ Failure Assessment
ssRNA	Single-stranded RNA
STING	Stimulator of interferon genes
TCS	Two-component system
TBK1	TANK-binding kinase 1
Th1	T helper 1
Th17	T helper 17
TLR	Toll-like receptor
TMPRSS2	Transmembrane protease serine 2
TNF-α	Tumor necrosis factor-α
TRIF	TIR-domain-containing adapter-inducing interferon-β
WHO	World Health Organization
